# Knowledge of the Association Between Periodontal Diseases and Adverse Pregnancy Outcomes among Pregnant Women in Ivory Coast: A Cross-Sectional Study

**DOI:** 10.3290/j.ohpd.b5883991

**Published:** 2024-12-17

**Authors:** Zocko Ange Désiré Pockpa, Gnaba Samson Mobio, Aboubacar Sidiki Thissé Kane, Nadin Thérèse Koffi-Coulibaly, Assem Soueidan, Camille Bechina, Xavier Struillou

**Affiliations:** a Zocko Ange Désiré Pockpa Assistant Professor, Department of Periodontology, Dental College, Felix Houphouet Boigny University, Abidjan, Côte d’Ivoire. Idea, study design, performed periodontal examinations, wrote the manuscript.; b Gnaba Samson Mobio Associate Professor, Department of Periodontology, Dental College, Felix Houphouet Boigny University, Abidjan, Côte d’Ivoire. Critical proofreading and discussion, read and approved the manuscript.; c Aboubacar Sidiki Thissé Kane Assistant Professor, Department of Odontology, Armed Forces Medical-Surgical Center, Bamako, Mali. Critical proofreading and discussion, read and approved the manuscript.; d Nadin Thérèse Koffi-Coulibaly Professor, Department of Periodontology, Dental College, Felix Houphouet Boigny University, Abidjan, Côte d’Ivoire. Project supervisor, study design, synthesis of results, read and approved the manuscript.; e Assem Soueidan Professor, Department of Periodontology, Dental College, University of Nantes, Nantes, France. Project supervisor, study design, synthesis of results, read and approved the manuscript.; f Camille Bechina Assistant Professor, Department of Periodontology, Dental College, University of Nantes, Nantes, France. Critical proofreading and discussion, read and approved the manuscript.; g Xavier Struillou Associate Professor, Department of Periodontology, Dental College, University of Nantes, Nantes, France. Project supervisor, study design, synthesis of results, read and approved the manuscript.

**Keywords:** attitude, knowledge, periodontal disease, pregnancy, pregnant women

## Abstract

**Purpose:**

Several studies have established a significant association between periodontal diseases and adverse pregnancy outcomes, such as preterm birth, low birth weight and preeclampsia. Despite this, awareness among pregnant women, particularly in developing countries, remains insufficient, potentially impeding the adoption of preventive measures. This study aimed to evaluate the knowledge of pregnant women in Ivory Coast regarding the association between periodontal diseases and adverse pregnancy outcomes.

**Materials and Methods:**

This cross-sectional study included pregnant women attending antenatal clinics in the gynecology-obstetrics department of the Cocody University Hospital in Ivory Coast. A questionnaire was distributed to assess sociodemographic characteristics and knowledge about the relationship between periodontal diseases and pregnancy complications. Data were analysed using the chi-squared test, with the level of significance set at p < 0.05.

**Results:**

The study sample included 338 pregnant women with an average age of 30.78 years (± 5.90). Of these, 24.26% were aware that periodontal disease could induce complications in pregnant women and newborns. The knowledge of pregnant women is correlated with their educational level (p = 0.023) and their profession (p = 0.009).

**Conclusion:**

Knowledge among pregnant women about the association between periodontal diseases and adverse pregnancy outcomes remains insufficient in Ivory Coast. These results highlight the necessity for continuous improvement of educational programs targeting pregnant women and healthcare providers on this topic.

Periodontal diseases, including gingivitis and periodontitis, are chronic, multifactorial inflammatory conditions associated with dysbiotic plaque biofilms and characterised by the destruction of the supporting structures of the teeth.^19,22^ Periodontal diseases affect a significant portion of the adult population worldwide.^19,21,22^ If left untreated, inflammatory processes in periodontal diseases can lead to tooth loss; these are independently associated with comorbidities and adverse pregnancy outcomes.^6,10,14,29,33^

Adverse pregnancy outcomes include a variety of complications that can occur during pregnancy, affecting both the mother and the fetus, such as preterm birth, low birth weight, preeclampsia, spontaneous abortion, and stillbirth.^4^ Several studies have shown a statistically significant association between periodontal diseases and adverse pregnancy outcomes.^6,23,28,33^ Indeed, periodontal diseases can release inflammatory mediators and bacteria into the bloodstream.^28^ These agents can reach the placenta, triggering an inflammatory response that could induce preterm birth or other obstetric complications.^6,9,10^ Thus, periodontal diseases in pregnant women can considerably increase the risk of these pregnancy complications, highlighting the importance of maintaining good oral health.^6^ Periodontal prevention and treatment could positively impact obstetric outcomes.^16^ Therefore, a shift in practices is important for both health professionals and expectant mothers. Nevertheless, it seems that knowledge and comprehension of periodontal diseases as a risk factor for pregnancy are insufficient among these populations.^2,29^

In Ivory Coast, access to information is influenced by various socioeconomic factors (level of education, income and employment, access to media, etc). However, having accurate information is the starting point for any practical change.^17,18^

The hypothesis of this study was that pregnant women in Ivory Coast have limited knowledge about the association between periodontal diseases and adverse pregnancy outcomes, and this lack of knowledge is influenced by several factors. This study aimed to evaluate the knowledge of the association between periodontal diseases and adverse pregnancy outcomes among pregnant women in Ivory Coast, noting that it is the first study of its kind conducted in Ivory Coast.

## MATERIALS AND METHODS

### Study Design and Setting

This cross-sectional study was conducted from January 2018 to December 2020 among pregnant women attending antenatal clinics in the gynecology-obstetrics department of the Cocody University Hospital in Ivory Coast.

### Study Participants and Sampling

The inclusion criteria were pregnant women who approved their participation in the research by written consent. The exclusion criteria included pregnant women who did not consent to participate, and those who did not understand French.

### Study Procedure

Patients received information regarding the purpose and procedures of the study. Upon confirmation of the inclusion criteria, eligible patients completed a structured questionnaire. This questionnaire had been pre-tested on a small sample to confirm the clarity and pertinence of the questions. The questionnaires were administered by a single trained investigator (PZ) during routine prenatal visits, collecting demographic information (age, education level, socio-economic status), knowledge of periodontal diseases (PD), and their potential impact on pregnancy and prenatal care practices (frequency of dental visits, oral hygiene practices, and advice received from healthcare providers about oral health).

### Data Analysis 

All analyses were conducted using SPSS software version 22 (SPSS; Chicago, IL, USA) with a significance threshold set at p < 0.05. Categorical variables were expressed as numbers (n) and percentage (%), and the chi-squared (χ^2^) test was used for their comparison between groups.

### Ethical Considerations

This study was approved by the Scientific Research Ethics Committee of the Odonto-stomatology Faculty of Felix Houphouet Boigny University in Ivory Coast (protocol number 31/2018). It was conducted in accordance with the Helsinki Declaration of 1975, as revised in 2013. All subjects were informed about the aim of the study and provided their verbal and written consent before being included in the study.

## RESULTS

The sample consisted of 338 pregnant women aged 15 to 50 years, with an average age of 30.78 ± 5.90 years. A higher level of education was reported, and the majority of the participants were either tradespeople/workers (36.69%) or senior managers (21.01%). Most were in their third trimester of pregnancy (47.34%) and had had their first prenatal consultation during the first trimester of pregnancy. A statistically significant majority, 92.4%, had not consulted a dentist since becoming pregnant. The primary reason for not visiting the dentist was a lack of recommendation (50.22%).

A majority (64.20%) reported brushing their teeth twice daily. However, 35.6% found maintaining good oral hygiene during pregnancy challenging, primarily due to fatigue (26.97%) and gum pain (16.85%). Additionally, 37.2% reported experiencing oral and dental problems since the start of their pregnancy, with the most common issues being bleeding gums (39.78%) and dental pain (40.86%).

In the sample, 83 women (24.26%) were aware that periodontal disease could induce complications in pregnant women and newborns. Most indicated that periodontal diseases could lead to difficult delivery (89.02%), miscarriage (84.15%), preterm birth (75.61%), and low birth weight (80.49%). None were aware of the link to preeclampsia. Knowledge of these risks was correlated with their level of education (p = 0.023) and their profession (p = 0.009). The main sources of information were dentists (43.09%) and the media (26.83%).

## DISCUSSION

The exploration of a link between periodontal disease and pregnancy outcomes in Ivory Coast was conducted for the first time via a cohort study of pregnant women (RAPIG study) in four phases: inclusion and questionnaire responses; periodontal examination; follow-up; and collection of obstetric data post-delivery.^24^ This article focuses on the questionnaire responses regarding the pregnant women’s awareness of the association between periodontal diseases and adverse pregnancy outcomes.

Several studies highlight a considerable gap in awareness regarding the impact of periodontal diseases on adverse pregnancy outcomes among pregnant women in many countries. ^2,3,11,26,27,29,30^ The present study underscored the limited knowledge of pregnant women about the association between periodontal diseases and adverse pregnancy outcomes. Only 24.26% were aware that periodontal disease could induce complications in pregnant women and newborns. These findings are consistent with previous studies where no more than 30% of pregnant women were aware of the association between periodontal diseases and adverse pregnancy outcomes.^3,26,27,29,30^

In Jordan, Alwaeli et al^3^ found that among 275 pregnant women, 5.1% believed in a possible association between periodontal diseases and preterm birth. George et al^11^ studied 241 pregnant women in South-West Sydney and discovered that only 10% had been informed about perinatal oral health, with over 50% being unaware of the potential impact of poor maternal oral health on pregnancy. Wu et al^30^ observed, based on data from 832 women (including 188 pregnant women), that 74% and 91% of pregnant and non-pregnant women, respectively, had little knowledge of an association between pregnancy and oral health.

Among the pregnant women who acknowledged that periodontal diseases can impact pregnancy outcomes, 20% to 25% had no clear idea of the nature of the obstetric complications associated with periodontal diseases. They often believed that the major complications included difficult labor or miscarriage. However, the literature indicates that the primary complications are actually preterm birth, low birth weight, and preeclampsia.^1,8,15,23^ It is essential to increase awareness among pregnant women about the importance of maintaining good oral hygiene, both before and particularly during pregnancy. The lack of awareness and sensitisation regarding periodontal diseases during pregnancy can significantly impact pregnancy outcomes. Educating pregnant women on oral health is vital, as it helps prevent complications for both mother and child.^24^ Intensifying awareness campaigns about oral health for pregnant women is very important. Indeed, studies have shown statistically significant improvements in oral health knowledge after awareness campaigns.^12^ These campaigns can be conducted via conventional and digital media, social networks, and by distributing educational materials such as brochures and videos during prenatal visits. Such initiatives would aid in educating pregnant women about the important of good oral hygiene and the necessity of regular dental check-ups. Additionally, healthcare providers, including obstetricians, midwives, and dentists, should become more involved in raising awareness. The main source of information regarding the relationship between periodontal diseases and adverse pregnancy outcomes in this study was dentists (29.52%). However, only 1.22% of women were informed of the relationship between periodontal diseases and pregnacy during their prenatal consultations with gynecologists or midwives. Previous studies have highlighted that, despite their knowledge, many gynecologists do not routinely incorporate oral health assessments into their practice.^5,7,13,25^ Obstetricians and midwives, being on the front line in monitoring pregnant women, should therefore be the primary source of information.

In this study, the knowledge of pregnant women was correlated with their educational level (p = 0.023) and their profession (p = 0.009). Similar findings were observed in studies performed in China^30^ and India.^27^ Indeed, educational level and socioeconomic status are two interconnected factors that play a crucial role in access to information. Quality education and high socioeconomic status not only facilitate access to information but also improve the ability to use and critically evaluate that information. Policies aimed at improving access to education and reducing socioeconomic inequalities are essential to ensuring equitable access to information for all.^29^

Within its limitations, this study provides initial insights into pregnant women’s awareness of the association between periodontal diseases and pregnancy in Ivory Coast. The results are in line with current research; future research will further elucidate the progression of knowledge levels among pregnant women in Ivory Coast. This observation highlights the need for increased efforts in both education and preventive dental care.

## CONCLUSION

Knowledge among pregnant women about the link between periodontal diseases and adverse pregnancy outcomes remains low in Ivory Coast. To enhance awareness on this topic, we recommend integrating oral health into prenatal care, training obstetricians and midwives, conducting public health campaigns, and improving access to dental care.

**Fig 1:**
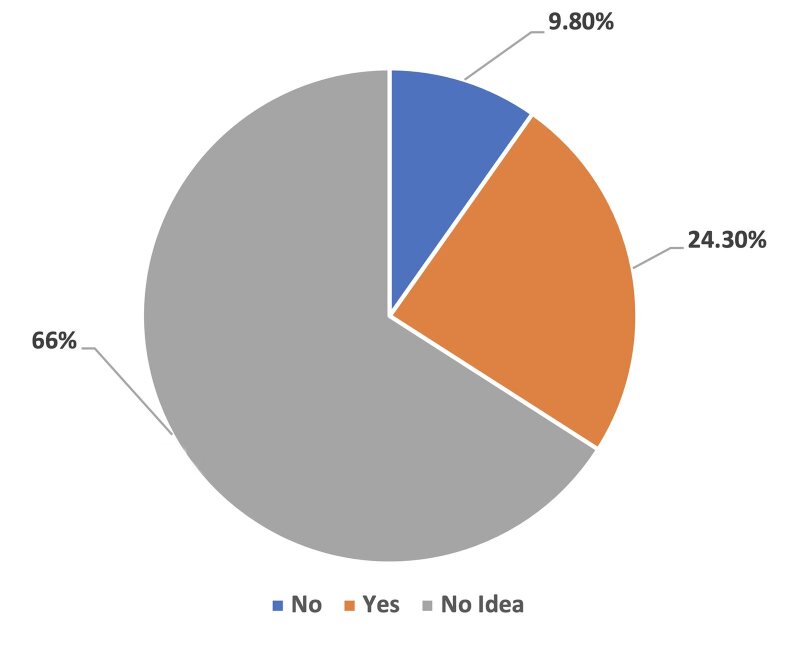
Distribution of pregnant women according to the knowledge of the association between periodontal diseases and adverse pregnancy outcomes or response to the question “Do you think PD represents a risk for the pregnant woman and her baby?”

**Table 1:** Sociodemographic characteristics and level of knowledge

Variable	p-value
Educational level	0.023*
Profession	0.009*
Age	0.175
Gestational age	0.509
Last dental visit	0.133
* Significant association.

**Fig 2:**
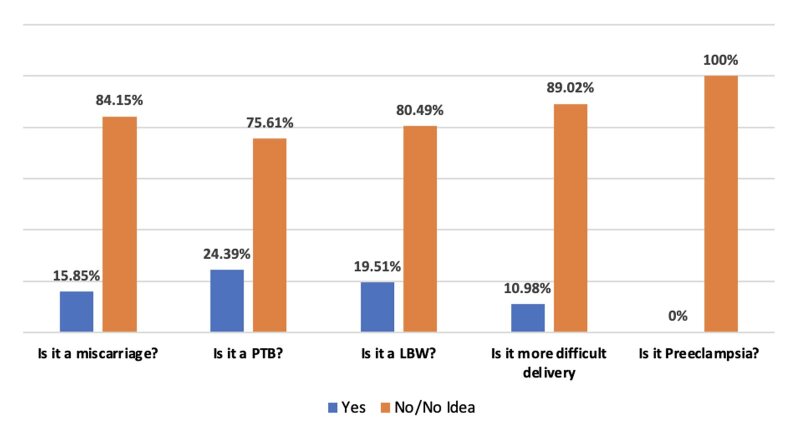
Distribution of informed pregnant women according to the adverse pregnancy outcomes associated with periodontal diseases or responses to thequestions: Is it a miscarriage? Preterm birth? Low birth weight? More difficult delivery? Preeclampsia? PTB: pre-term birth; LBW: low birth weight.

**Fig 3:**
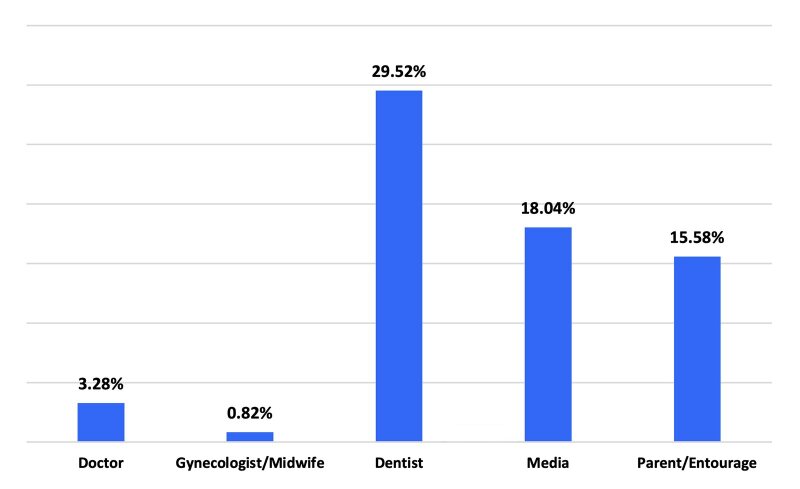
Distribution of pregnant women according to the source of information or response to the question “If yes, how did you obtain this information?”
